# Jagged1-mediated myeloid Notch1 signaling activates HSF1/Snail and controls NLRP3 inflammasome activation in liver inflammatory injury

**DOI:** 10.1038/s41423-019-0318-x

**Published:** 2019-10-31

**Authors:** Yuting Jin, Changyong Li, Dongwei Xu, Jianjun Zhu, Song Wei, Andrew Zhong, Mingwei Sheng, Sergio Duarte, Ana J. Coito, Ronald W. Busuttil, Qiang Xia, Jerzy W. Kupiec-Weglinski, Bibo Ke

**Affiliations:** 1 The Dumont-UCLA Transplant Center, Division of Liver and Pancreas Transplantation, Department of Surgery, David Geffen School of Medicine at UCLA, Los Angeles, CA USA; 2 Department of Liver Surgery, Renji Hospital, Shanghai Jiaotong University School of Medicine, Shanghai, China; 3 Department of Physiology, School of Basic Medical Sciences, Wuhan University, Wuhan, China

**Keywords:** Jagged1, Notch1, NLRP3, Innate immunity, Liver injury, Immunology, Cell biology

## Abstract

Notch signaling plays important roles in the regulation of immune cell functioning during the inflammatory response. Activation of the innate immune signaling receptor NLRP3 promotes inflammation in injured tissue. However, it remains unknown whether Jagged1 (JAG1)-mediated myeloid Notch1 signaling regulates NLRP3 function in acute liver injury. Here, we report that myeloid Notch1 signaling regulates the NLRP3-driven inflammatory response in ischemia/reperfusion (IR)-induced liver injury. In a mouse model of liver IR injury, Notch1-proficient (Notch1^FL/FL^) mice receiving recombinant JAG1 showed a reduction in IR-induced liver injury and increased Notch intracellular domain (NICD) and heat shock transcription factor 1 (HSF1) expression, whereas myeloid-specific Notch1 knockout (Notch1^M-KO^) aggravated hepatocellular damage even with concomitant JAG1 treatment. Compared to JAG1-treated Notch1^FL/FL^ controls, Notch1^M-KO^ mice showed diminished HSF1 and Snail activity but augmented NLRP3/caspase-1 activity in ischemic liver. The disruption of HSF1 reduced Snail activation and enhanced NLRP3 activation, while the adoptive transfer of HSF1-expressing macrophages to Notch1^M-KO^ mice augmented Snail activation and mitigated IR-triggered liver inflammation. Moreover, the knockdown of Snail in JAG1-treated Notch1^FL/FL^ livers worsened hepatocellular functioning, reduced TRX1 expression and increased TXNIP/NLRP3 expression. Ablation of myeloid Notch1 or Snail increased ASK1 activation and hepatocellular apoptosis, whereas the activation of Snail increased TRX1 expression and reduced TXNIP, NLRP3/caspase-1, and ROS production. Our findings demonstrated that JAG1-mediated myeloid Notch1 signaling promotes HSF1 and Snail activation, which in turn inhibits NLRP3 function and hepatocellular apoptosis leading to the alleviation of IR-induced liver injury. Hence, the Notch1/HSF1/Snail signaling axis represents a novel regulator of and a potential therapeutic target for liver inflammatory injury.

## Introduction

Hepatic ischemia/reperfusion injury (IRI) is one of the leading causes of hepatocellular dysfunction or failure during liver transplantation or resection. Hepatic IRI activates liver macrophages (Kupffer cells) and promotes the production of reactive oxygen species (ROS) and proinflammatory cytokines, including TNF-*α* and IL-1, to trigger a sterile inflammatory response.^1^ We have demonstrated the importance of NLRP3 function in the mechanism of liver IRI.^2^ Indeed, as an innate immune signaling receptor, NLRP3 is a key danger signal sensor that drives the inflammatory response in IR-stressed liver.

The Snail family of transcription factors consists of Snail1 (Snail), Snail2 (Slug) and Snail3 (Smuc), which share an evolutionarily conserved role in the processes of cell development and differentiation.^3^ Snail not only acts primarily as a key regulator of the epithelial−mesenchymal transition but also contributes to cell survival.^4,5^ Activation of Snail induces anti-inflammatory cytokines and modulates immune responses.^6^ During tissue inflammation and injury, Snail promotes wound healing through regulation of TGF-β signaling.^7^ Moreover, Snail regulates cell growth via an ROS-mediated pathway. Disruption of Snail signaling was shown to increase ROS production to cause reduced cell survival under oxidative stress,^8^ suggesting that Snail may play an important role in the modulation of ROS-mediated inflammation.

Four different Notch receptors (Notch1−4) and five Notch ligands (Jagged1, Jagged2, DLL1, DLL3, and DLL4) have been described in mammals.^9^ Binding of l proteins to the Notch extracellular domain induce proteolytic cleavage and the release of the Notch intracellular domain (NICD), which binds to the nuclear recombinant recognition sequence binding protein at the Jκ site (RBP-J) to activate Notch target genes.^10^ Jagged1 (JAG1) is required for the control of cell development.^11^ Activation of JAG1-mediated Notch1 signaling regulates smooth muscle differentiation and blood vessel formation.^12^ Increasing the expression of Notch1 and its ligand JAG1 contributes to cell growth and survival during liver regeneration.^13^ Moreover, Notch signaling controls innate immune cell homeostasis and function.^14^ Disruption of the transcription factor RBP-J increases cell apoptosis/necrosis and inflammatory responses, resulting in exacerbated liver inflammatory injury.^15^ Although these studies have shown that Notch signaling is correlated with hepatocellular protection during liver inflammation, it remains largely unknown whether and how JAG1-mediated Notch signaling may regulate the NLRP3-driven inflammatory response in liver IRI.

Here, we identified the novel functional role and regulatory mechanism of JAG1-mediated myeloid Notch1 signaling in liver sterile inflammatory injury. We demonstrate that myeloid Notch1 signaling promotes heat shock transcription factor 1 (HSF1) and Snail activation, which in turn controls NLRP3-mediated innate immunity during IR-triggered liver inflammation.

## Results

### JAG1-mediated myeloid Notch1 signaling reduces hepatocellular damage in IR-stressed liver

**Fig. 1:**
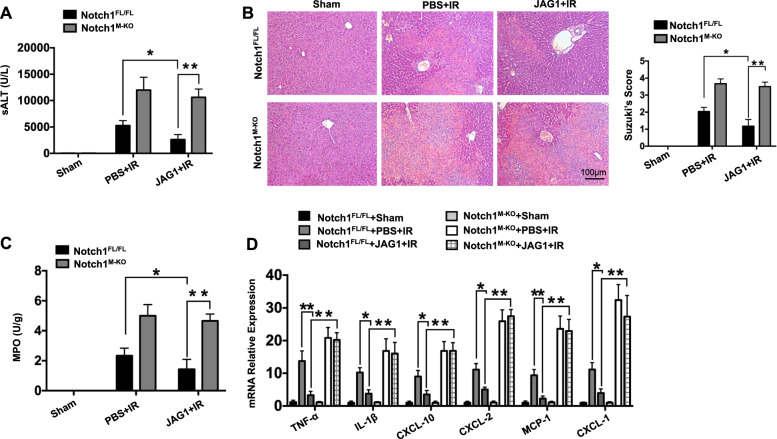
JAG1-mediated myeloid Notch1 signaling reduces hepatocellular damage in IR-stressed liver. Notch1^FL/FL^ and Notch1^M-KO^ mice were treated with recombinant JAG1 (0.5 mg/kg) or PBS and then subjected to 90 min of warm ischemia followed by 6 h of reperfusion. **a** Liver function was evaluated according to the serum ALT (sALT) levels (IU/L) (*n* = 4–6 samples/group). **b** Representative histological staining (H&E) of ischemic liver tissue (*n* = 4–6 mice/group) and the Suzuki histological score. Scale bars, 100 μm. **c** Liver neutrophil accumulation, as analyzed according to MPO activity (U/g) (*n* = 4–6 samples/group). **d** qRT-PCR-assisted detection of TNF-α, IL-1β, CXCL-10, CXCL-2, MCP-1, and CXCL-1 in ischemic livers (*n* = 3-4 samples/group). Data were normalized to HPRT gene expression. All data represent the mean ± SD. *p < 0.05, **p < 0.01

Myeloid-specific Notch1-deficient (Notch1^M-KO^) and Notch1-proficient (Notch1^FL/FL^) mice treated with recombinant JAG1 or phosphate buffer saline (PBS) were subjected to liver IR (90 min). After 6 h of reperfusion, JAG1 treatment significantly (*p* < 0.05) reduced sALT levels in the Notch1^FL/FL^ mice compared to those in the PBS-treated controls (Fig. 1a). In contrast, the disruption of myeloid Notch1 signaling increased sALT levels in Notch1^M-KO^ mice with or without JAG1 treatment. These data correlated with Suzuki’s histological grading of liver IRI (Fig. 1b). Unlike the PBS-treated Notch1^FL/FL^ controls, which showed moderate to severe edema, sinusoidal congestion, and necrosis after liver IRI, the JAG1-treated Notch1^FL/FL^ mice were characterized by reduced edema and sinusoidal congestion and mild necrosis. However, the Notch1^M-KO^ mice showed much more advanced histological damage even after concomitant JAG1 treatment. The JAG1-treated Notch1^FL/FL^ livers showed significantly (*p* < 0.05) decreased MPO levels, whereas Notch1^M-KO^ increased MPO levels (Fig. 1c) and the mRNA expression of TNF-α, IL-1β, CXCL-10, CXCL-2, MCP1, and CXCL-1 (Fig. 1d).

### JAG1-mediated myeloid Notch1 signaling promotes HSF1/Snail activation and inhibits TXNIP/NLRP3 activation and reduces macrophage/neutrophil infiltration in IR-stressed liver

**Fig. 2:**
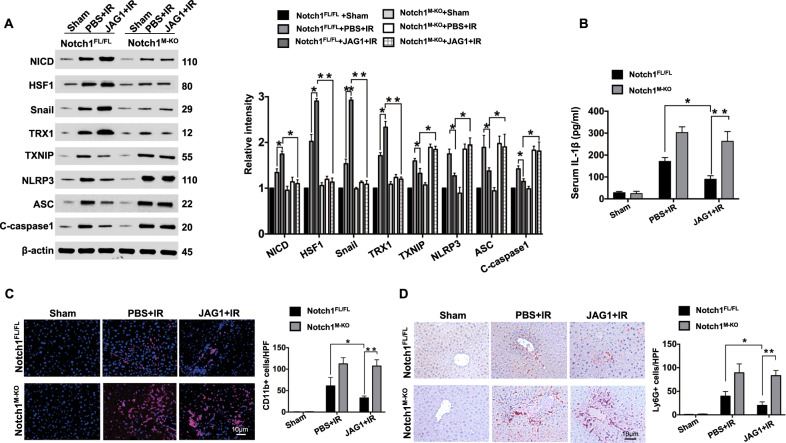
JAG1-mediated myeloid Notch1 signaling promotes HSF1/Snail activation and inhibits TXNIP/NLRP3 activation with reduced macrophage/neutrophil infiltration in IR-stressed liver. **a** Western blot-assisted analysis and relative density ratios of NICD, HSF1, Snail, TRX1, TXNIP, NLRP3, ASC, and cleaved caspase-1 expression in IR-stressed livers. Representative of three experiments. **b** ELISA analysis of serum IL-1β levels (*n* = 3−4 samples/group). **c** Immunofluorescence staining of CD11b^+^ macrophages in ischemic livers (*n* = 4–6 mice/group). Quantification of CD11b^+^ macrophages; scale bars, 10 μm. **d** Immunohistochemical staining of Ly6G^+^ neutrophils in ischemic livers (*n* = 4–6 mice/group). Quantification of Ly6G^+^ neutrophils; scale bars, 10 μm. All data represent the mean ± SD. *p < 0.05, **p < 0.01

We then asked whether JAG1-mediated myeloid Notch1 signaling may influence HSF1 and Snail activation in IR-stressed liver. JAG1 treatment in the ^/FL^ livers increased NICD, HSF1, Snail, and thioredoxin (TRX)1 expression but reduced thioredoxin-interacting protein (TXNIP), NLRP3, ASC, and cleaved caspase-1 protein expression compared to that in PBS-treated controls (Fig. 2a). However, Notch1^M-KO^ with or without JAG1 treatment diminished NICD, HSF1, Snail, and TRX1 activation and enhanced TXNIP, NLRP3, ASC, and cleaved caspase-1 activation (Fig. 2a), leading to significantly (*p* < 0.01) increased IL-1β release (Fig. 2b). The JAG1-treated Notch1^FL/FL^ livers showed reduced CD11b^+^ macrophage (Fig. 2c) and Ly6G^+^ neutrophil trafficking (Fig. 2d) compared to that in PBS-treated controls, whereas Notch1^M-KO^ increased CD11b^+^ macrophage (Fig. 2c) and Ly6G^+^ neutrophil accumulation (Fig. 2d) even in the presence of JAG1 treatment.

### JAG1-mediated myeloid Notch1 signaling reduces hepatocellular apoptosis/necrosis in IR-stressed liver

**Fig. 3:**
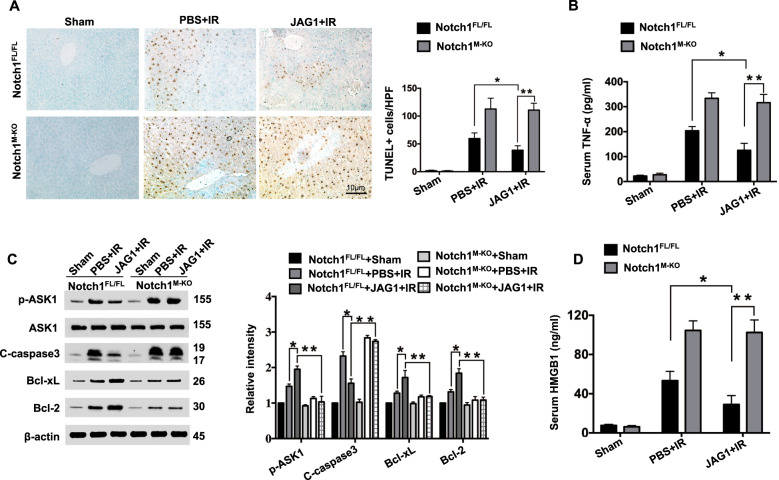
JAG1-mediated myeloid Notch1 signaling reduces hepatocellular apoptosis/necrosis in IR-stressed liver. **a** Liver apoptosis according to TUNEL staining in ischemic mouse livers (*n* = 4–6 mice/group). Quantification of apoptotic cells; scale bars, 10 μm. **b** ELISA analysis of serum TNF-α levels (*n* = 3−4 samples/group). **c** Western-blotting-assisted analysis and relative density ratios of p-ASK1, cleaved caspase-3, Bcl-2, and Bcl-xL expression. Representative of three experiments. **d** ELISA analysis of serum HMGB1 levels (*n* = 3−4 samples/group). All data represent the mean ± SD. *p < 0.05, **p < 0.01

To determine whether the activation of JAG1-mediated myeloid Notch1 signaling may regulate cell apoptosis in response to liver IRI, we performed TUNEL staining to analyze IR-induced hepatocellular apoptosis in ischemic livers. Indeed, livers in JAG1-treated Notch1^FL/FL^ mice showed a reduced number of apoptotic TUNEL^+^ cells compared to that in PBS-treated controls (Fig. 3a). However, the number of TUNEL^+^ cells was significantly (*p* < 0.01) increased in the Notch1^M-KO^ mice even with concomitant JAG1 treatment (Fig. 3a). An ELISA assay revealed that JAG1 treatment significantly (*p* < 0.01) reduced serum TNF-α production in the Notch1^FL/FL^ mice, whereas Notch1^M-KO^ mice showed increased TNF-α production (Fig. 3b). Western blot analysis revealed that JAG1 treatment in the Notch1^FL/FL^ livers upregulated Bcl-2 and Bcl-xL expression but downregulated p-ASK1 and cleaved caspase-3 expression compared to that in PBS-treated controls (Fig. 3c). In contrast, Notch1^M-KO^ livers showed reduced Bcl-2 and Bcl-xL expression but augmented p-ASK1 and cleaved caspase-3 expression (Fig. 3c). Moreover, increased HMGB1 production was observed in the Notch1^M-KO^ mice but not in the JAG1-treated Notch1^FL/FL^ mice (Fig. 3d).

### Myeloid Notch1 signaling-induced HSF1 expression activates Snail and regulates NLRP3-mediated immune responses in IR-stressed liver

**Fig. 4:**
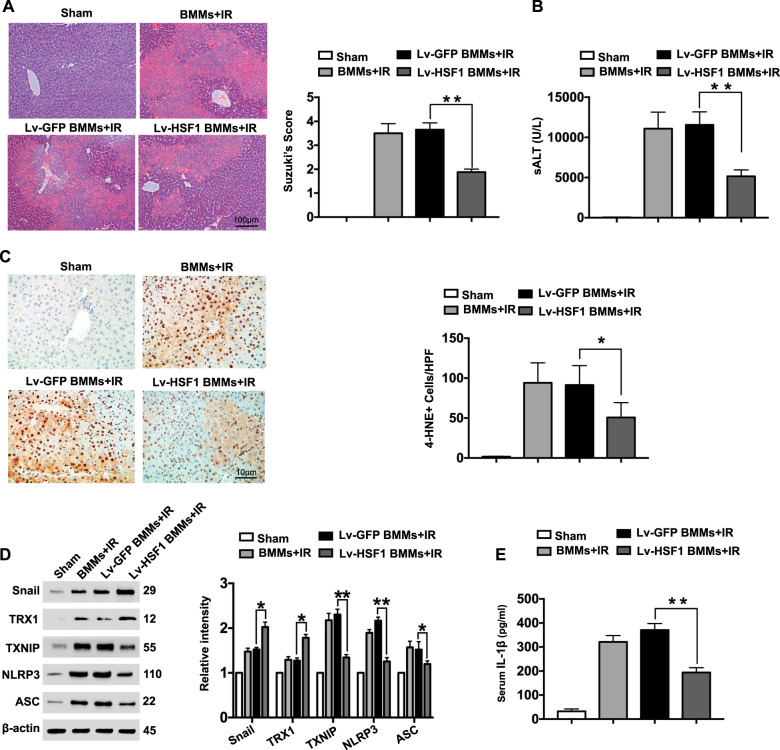
Myeloid Notch1 signaling-induced HSF1 expression activates Snail and regulates the NLRP3-mediated immune response in IR-stressed liver. Notch1^M-KO^ mice were injected via the tail vein with bone marrow-derived macrophages (BMMs, 5 × 10^6^ cells/mouse) transfected with lentivirus expressing HSF1 (Lv-HSF1) or GFP control (Lv-GFP) 24 h prior to ischemia. **a** Representative histological staining (H&E) of ischemic liver tissue (*n* = 4–6 mice/group) and the Suzuki histological score. Scale bars, 100 μm. **b** sALT levels (IU/L) (*n* = 4–6 samples/group). **c** Immunohistochemistry staining of 4-HNE^+^ cells in ischemic livers (*n* = 4–6 mice/group). Quantification of 4-HNE^+^ cells; scale bars, 10 μm. **d** Western-blotting-assisted analysis and relative density ratios of Snail, TRX1, TXNIP, NLRP3, and ASC expression. Representative of three experiments. **e** ELISA analysis of serum IL-1β levels (*n* = 3−4 samples/group). All data represent the mean ± SD. *p < 0.05, **p < 0.01

Next, we evaluated the functional role of myeloid Notch1 signaling-induced HSF1 expression in IR-stressed liver. BMMs were transfected with lentivirus expressing HSF1 (Lv-HSF1) or a GFP control (Lv-GFP) and adoptively transferred into Notch1^M-KO^ mice. Unlike livers treated with Lv-GFP-transfected BMMs or control cells, livers in Notch1^M-KO^ mice treated with Lv-HSF1-transfected cells showed a reduction in IR-induced hepatocellular damage, as evidenced by the decrease in the Suzuki histological score (Fig. 4a) and sALT levels (Fig. 4b). Moreover, the adoptive transfer of Lv-HSF1-transfected cells into the Notch1^M-KO^ ischemic livers decreased the cellular expression of 4-hydroxynonenal (4-HNE), a marker of ROS production^16^ (Fig. 4c). Liver treated with Lv-HSF1-transfected cells showed increased Snail and TRX1 expression but reduced TXNIP, NLRP3, and ASC expression (Fig. 4d), which was accompanied by diminished IL-1β release (Fig. 4e) compared to that in the Lv-GFP-treated group or cell controls.

### Myeloid Notch1 signaling controls NLRP3 function in a Snail-dependent manner in IR-stressed liver

**Fig. 5:**
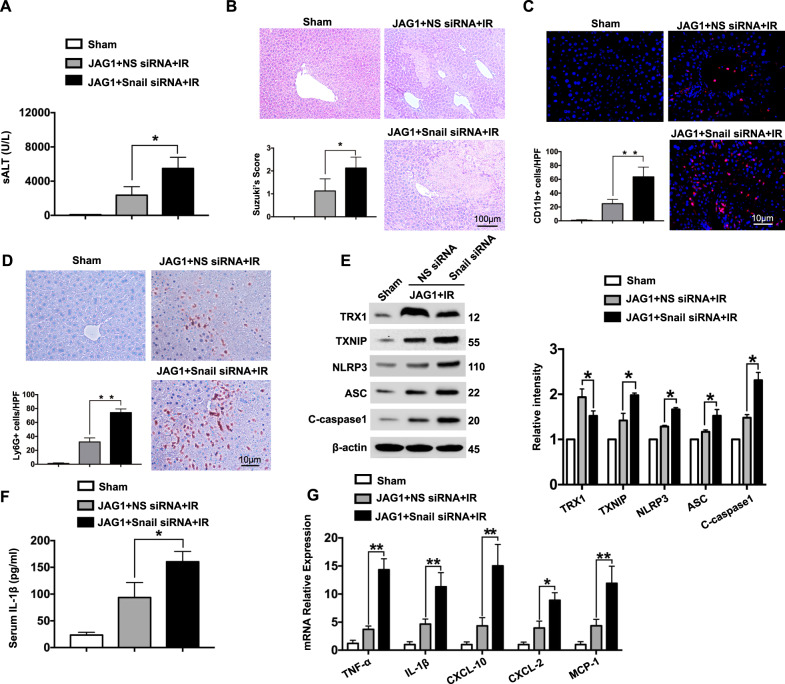
Myeloid Notch1 signaling controls NLRP3 functioning in a Snail-dependent manner in IR-stressed liver. Notch1^FL/FL^ mice were injected via the tail vein with Snail siRNA (2 mg/kg) or nonspecific (NS) control siRNAs mixed with mannose-conjugated polymers 4 h prior to ischemia. **a** sALT levels (IU/L) (*n* = 4–6 samples/group). **b** Representative histological staining (H&E) of ischemic liver tissue (*n* = 4–6 mice/group) and the Suzuki histological score. Scale bars, 100 μm. **c** Immunofluorescence staining of CD11b^+^ macrophages in ischemic livers (*n* = 4–6 mice/group). Quantification of CD11b^+^ macrophages; scale bars, 10 μm. **d** Immunohistochemical staining of Ly6G^+^ neutrophils in ischemic livers (*n* = 4–6 mice/group). Quantification of Ly6G^+^ neutrophils; scale bars, 10 μm. **e** Western blot analysis and relative density ratios of TRX1, TXNIP, NLRP3, ASC, and cleaved caspase-1 expression. Representative of three experiments. **f** ELISA analysis of serum IL-1β levels (*n* = 3−4 samples/group). **g** Quantitative RT-PCR-assisted detection of mRNAs encoding TNF-α, IL-1β, CXCL-10, CXCL-2, and MCP-1 (*n* = 3−4 samples/group). All data represent the mean ± SD. *p < 0.05, **p < 0.01

As myeloid Notch1-induced HSF1 signaling promoted Snail activation in IR-stressed liver, we then asked whether Snail is required for the regulation of NLRP3 activation in response to liver IRI. We disrupted Snail in Notch1^FL/FL^ livers with an in vivo mannose-mediated Snail siRNA delivery system specifically targeted to macrophages.^2^ Unlike the administration of nonspecific (NS) siRNA in the JAG1-treated Notch1^FL/FL^ mice, the knockdown of Snail significantly (*p* < 0.05) increased the sALT levels (Fig. 5a) and IR-induced liver damage (Fig. 5b) in the JAG1-treated Notch1^FL/FL^ mice. Snail siRNA treatment in the JAG1-treated Notch1^FL/FL^ livers increased CD11b^+^ macrophage (Fig. 5c) and neutrophil (Fig. 5d) accumulation. Moreover, Snail knockdown reduced TRX1 expression but augmented TXNIP, NLRP3, ASC, and cleaved caspase-1 expression (Fig. 5e), leading to increased IL-1β production (Fig. 5f) and TNF-α, IL-1β, CXCL-10, CXCL-2, and MCP1 expression (Fig. 5g).

### Notch1 signaling-induced HSF1 expression promotes Snail activation and inhibits NLRP3 activation in macrophages

**Fig. 6:**
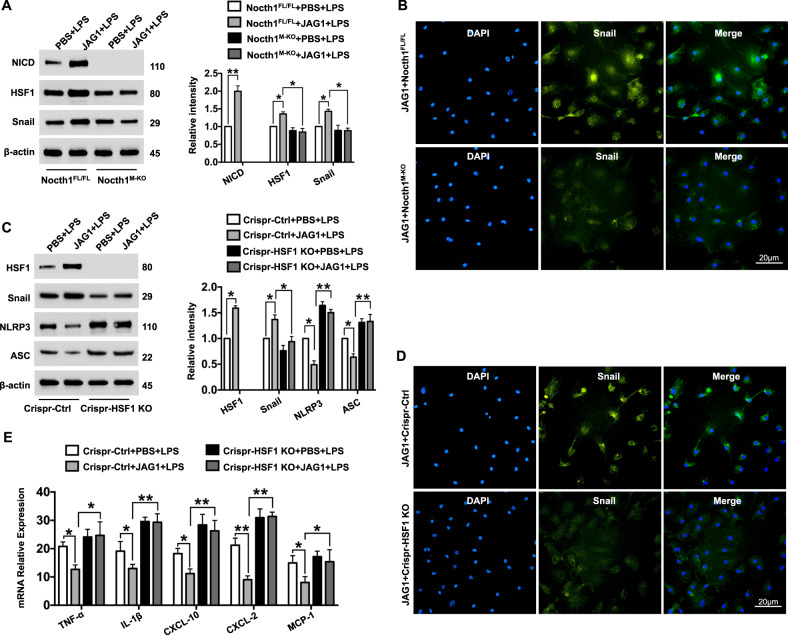
Notch1 signaling-induced HSF1 expression promotes Snail activation and inhibits NLRP3 activation in macrophages. BMMs were isolated from Notch1^FL/FL^ and Notch1^M-KO^ mice and treated with JAG1 (2 µg/ml) followed by 6 h of LPS (100 ng/ml) stimulation. **a** Western blot analysis and relative density ratios of NICD, HSF1, and Snail expression. Representative of three experiments. **b** Representative immunofluorescence staining of Snail expression in macrophages (*n* = 3−4 samples/group). DAPI was used to visualize the nuclei. Scale bars, 20 μm. **c** BMMs (1 × 10^6^/well) from Notch1^FL/FL^ mice were transfected with p-CRISPR-HSF1 KO or control vector and then treated with JAG1 followed by 6 h of LPS stimulation. Western blot analysis and relative density ratios of HSF1, Snail, NLRP3, and ASC expression. Representative of three experiments. **d** Representative immunofluorescence staining of Snail expression in macrophages (*n* = 3−4 samples/group). DAPI was used to visualize the nuclei. Scale bars, 20 μm. **e** Quantitative RT-PCR-assisted detection of mRNAs encoding TNF-α, IL-1β, CXCL-10, CXCL-2, and MCP-1 (*n* = 3−4 samples/group). All data represent the mean ± SD. *p < 0.05, **p < 0.01

Having demonstrated the importance of JAG1-mediated myeloid Notch1 signaling in the regulation of HSF1/Snail and NLRP3 function in vivo, we next tested whether there was crosstalk between Notch1 signaling-induced HSF1 and Snail that affected the control of NLRP3-mediated immune responses selectively in macrophages. We found increased NICD and HSF1 protein expression in Notch1^FL/FL^ macrophages after JAG1 treatment (Fig. 6a). Notably, JAG1-treated Notch1^FL/FL^ macrophages showed increased Snail protein expression, while Notch1^M-KO^ inhibited HSF1 and Snail expression (Fig. 6a). Immunofluorescence staining revealed increased Snail expression in Notch1^FL/FL^ but not in Notch1^M-KO^ macrophages after JAG1 treatment (Fig. 6b). Moreover, the transfection of Notch1^FL/FL^ macrophages with p-CRISPR-HSF1 KO reduced Snail expression but augmented NLRP3 and ASC expression compared to that in the control vector-treated cells (Fig. 6c). This was further confirmed by the immunofluorescence staining data, which showed that p-CRISPR-HSF1 KO reduced Snail expression in JAG1-treated macrophages (Fig. 6d), which was accompanied by the increased mRNA expression of TNF-α, IL-1β, CXCL-10, CXCL-2, and MCP1 (Fig. 6e).

### Snail is crucial for the Notch1 signaling-mediated immune regulation of NLRP3 activation in macrophages

**Fig. 7:**
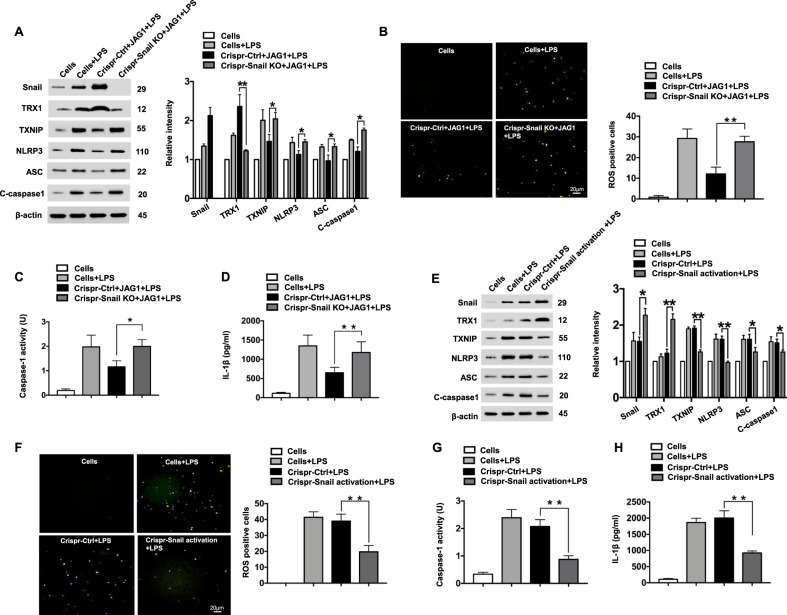
Snail is crucial for the Notch1 signaling-mediated immune regulation of NLRP3 activation in macrophages. BMMs (1 × 10^6^/well) from Notch1^FL/FL^ mice were transfected with p-CRISPR-Snail KO or control vector and then treated with JAG1 followed by 6 h of LPS stimulation. **a** Western blot analysis and relative density ratios of Snail, TRX1, TXNIP, NLRP3, ASC, and cleaved caspase-1 expression. Representative of three experiments. **b** ROS production was detected by Carboxy-H2DFFDA in LPS-stimulated BMMs from Notch1^FL/FL^ mice. Quantification of ROS-producing BMMs (green); scale bars, 20 μm. **c** Caspase-1 activity (U) (*n* = 3−4 samples/group). **d** ELISA analysis of supernatant IL-1β levels (*n* = 3−4 samples/group). **e** BMMs from Notch1^M-KO^ mice were transfected with p-CRISPR-Snail activation or control vector followed by 6 h of LPS stimulation. Western-blotting-assisted analysis and relative density ratios of Snail, TRX1, TXNIP, NLRP3, ASC, and cleaved caspase-1 expression. Representative of three experiments. **f** ROS production in LPS-stimulated BMMs from Notch1^M-KO^ mice. Quantification of ROS-producing BMMs (green). Scale bars, 20 μm. **g** Caspase-1 activity (U) (*n* = 3−4 samples/group). **h** ELISA analysis of supernatant IL-1β levels (*n* = 3−4 samples/group). All data represent the mean ± SD. *p < 0.05, **p < 0.01

To elucidate the mechanistic role of Snail in the regulation of NLRP3 function in Notch1 signaling-mediated immune regulation, BMMs from Notch1^FL/FL^ and Notch1^M-KO^ mice were transfected with the p-CRISPR-Snail KO, p-CRISPR-Snail or control vector followed by JAG1 and LPS treatment. Clearly, unlike control vector treatment, transfection of JAG1-treated Notch1^FL/FL^ cells with p-CRISPR Snail KO diminished TRX1 expression but markedly increased TXNIP, NLRP3, ASC, and cleaved caspase-1 expression (Fig. 7a) accompanied by augmented ROS production (Fig. 7b), caspase-1 activity (Fig. 7c), and IL-1β release (Fig. 7d). In contrast, the induction of Snail by transfecting Notch1^M-KO^ cells with p-CRISPR-Snail resulted in increased TRX1 expression and reduced TXNIP, NLRP3, ASC, and cleaved caspase-1 expression (Fig. 7e), ROS production (Fig. 7f), caspase-1 activity (Fig. 7g), and IL-1β release (Fig. 7h) compared to that in control vector-treated cells after LPS stimulation.

### Snail is essential for the regulation of hepatocellular apoptosis/necrosis during Notch1 signaling-mediated immune regulation

**Fig. 8:**
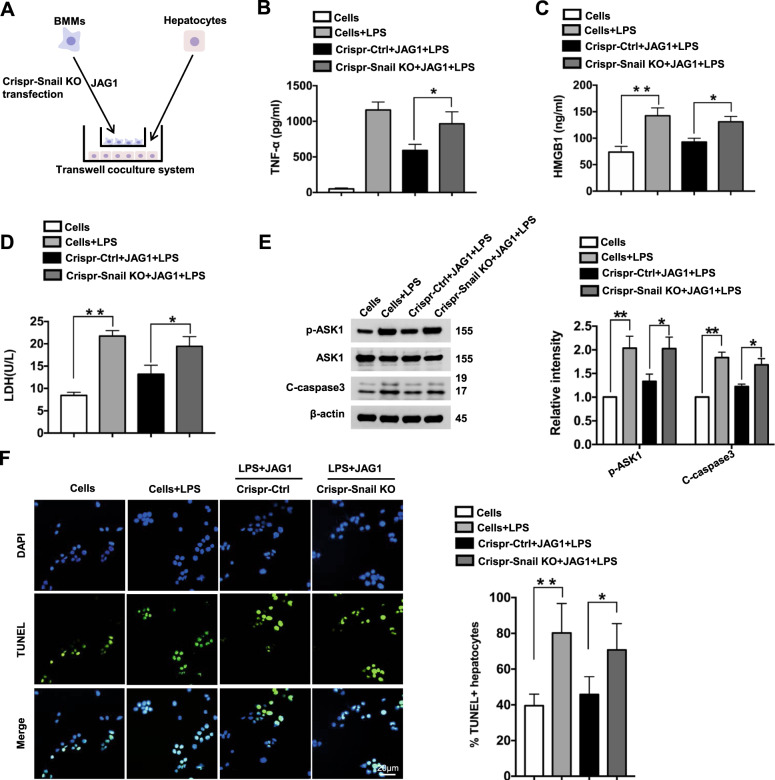
Snail is essential for the regulation of hepatocellular apoptosis/necrosis during Notch1 signaling-mediated immune regulation. **a** Schematic showing the macrophage/hepatocyte coculture system. **b** BMMs (1 × 10^6^) were isolated from Notch1^FL/FL^ mice and transfected with the p-CRISPR-Snail KO or control vector followed by JAG1 and LPS treatment and then cocultured with primary hepatocytes (4 × 10^5^/well) for 12 h with or without H_2_O_2_ (200 µM). ELISA analysis of supernatant TNF-α (**b**) and HMGB1 (**c**) levels in cocultures (*n* = 3−4 samples/group). **d** LDH release (U/L) from hepatocytes after coculture (*n* = 3−4 samples/group). **e** Western-blotting-assisted analysis and relative density ratios of p-ASK1 and cleaved caspase-3 expression in hepatocytes after coculture. Representative of three experiments. **f** Immunofluorescence staining of TUNEL^+^ hepatocytes after coculture (*n* = 4–6 mice/group). Quantification of TUNEL^+^ cells; scale bars, 20 μm. All data represent the mean ± SD. *p < 0.05, **p < 0.01

To dissect the molecular mechanism of JAG1-mediated Notch1 signaling involved in the modulation of hepatocyte apoptosis/necrosis during liver IRI, we used a BMM/hepatocyte coculture system (Fig. 8a). BMMs from Notch1^FL/FL^ mice were transfected with p-CRISPR-Snail KO or a control vector followed by JAG1 and LPS treatment and then cocultured with hepatocytes supplemented with H_2_O_2_. We found that BMMs transfected with p-CRISPR Snail KO showed markedly increased TNF-α (Fig. 8b) and HMGB1 (Fig. 8c) release compared to that of the control vector-treated groups in the coculture supernatants. Interestingly, increased LDH release from stressed hepatocytes was observed after coculture with CRISPR Snail KO-BMMs but not with control-treated cells (Fig. 8d). The expression of p-ASK1 and cleaved caspase-3 in stressed hepatocytes was significantly (*p* < 0.05) increased after coculture with CRISPR Snail KO-BMMs (Fig. 8e). Moreover, immunofluorescence staining revealed an increased number of apoptotic TUNEL^+^ hepatocytes after coculture with CRISPR Snail KO-BMMs compared with that in the control-treated groups (Fig. 8f).

## Discussion

**Fig. 9:**
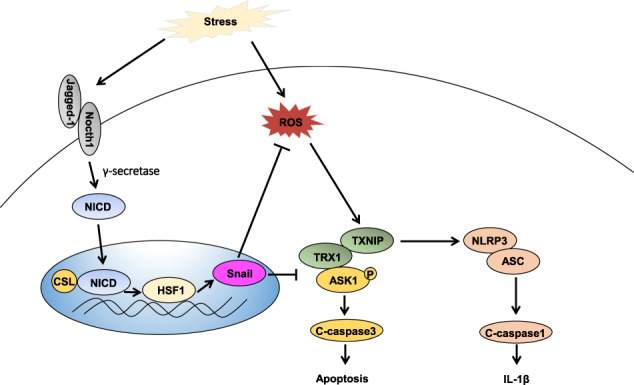
Schematic illustration of JAG1-mediated myeloid Notch1/HSF1/Snail signaling in the regulation of innate immune responses during liver IRI. IR stress activates Notch1 signaling and induces ROS production in ischemic livers. Upon ligand binding, Notch1 is cleaved by γ-secretase and releases the intracellular domain (NICD), which translocates into the nucleus and forms a complex with the CSL DNA-binding protein to activate HSF1; HSF1, in turn, promotes Snail activation and regulates the TRX1/TXNIP and TRX1/ASK1 complexes, leading to reduced NLRP3 inflammasome activation and ROS-induced hepatocellular apoptosis/necrosis in IR-triggered liver inflammation

To the best of our knowledge, this study is the first to document that JAG1-mediated myeloid Notch1/HSF1/Snail signaling is crucial for modulating NLRP3-mediated immune responses in response to IR-triggered liver inflammation. The principal findings are as follows: (i) JAG1-mediated myeloid Notch1 signaling alleviates IR-induced liver damage and reduces macrophage/neutrophil trafficking and proinflammatory mediator expression; (ii) JAG1-mediated myeloid Notch1 signaling promotes HSF1/Snail activation and inhibits NLRP3/caspase-1 activity; (iii) JAG1-mediated myeloid Notch1 signaling promotes antiapoptotic functioning and inhibits proapoptotic activity during IR-induced hepatocyte apoptosis; (iv) JAG1-mediated myeloid Notch1 signaling controls NLRP3 function and hepatocellular apoptosis via a Snail-dependent pathway. Our results highlight the importance of JAG1-mediated myeloid Notch1/HSF1/Snail signaling as a key regulator of NLRP3 activation and cell apoptosis during IR stress-mediated liver inflammation (Fig. 9).

The Notch pathway is one of the most frequently activated signaling pathways in human liver diseases. Since Notch signaling regulates multiple cell activities, the deregulation of the Notch cascade has been found to contribute to many pathological processes.^10^ The activation of Notch signaling by TLR4 stimulation can regulate gene expression patterns involved in proinflammatory responses.^17^ However, JAG1-mediated Notch1 signaling induces regulatory T-cell (Treg) production and exerts an immunoregulatory effect, whereas blockade of JAG1/Notch1 signaling inhibits Treg suppressor function both in vitro and in vivo*.*^18^ Induction of the Notch intracellular domain (NICD) suppresses proinflammatory cytokine expression while enhancing anti-inflammatory mediator expression in TLR-stimulated macrophages.^19^ Moreover, Notch1 and its target gene *Hes1* can inhibit the inflammatory response via an inhibitory feedback loop and transcriptional regulation.^20,21^ Disruption of myeloid Notch1 activity activates RhoA/ROCK signaling and exacerbates liver inflammation.^22^ These results suggest that Notch signaling modulates the inflammatory response through multiple mechanisms. The present study characterized a molecular circuit responsible for the regulatory effect of JAG1-mediated myeloid Notch1 signaling on NLRP3 function during liver IRI. We found that JAG1-mediated myeloid Notch1 signaling activated HSF1 and Snail, which in turn inhibited NLRP3 activation leading to reduced IR-induced liver inflammation. Our findings reveal a novel mechanism in which JAG1-mediated myeloid Notch1 signaling regulates the NLRP3-mediated innate immune response in IR-stressed livers.

HSF1 is the master transcriptional regulator of cellular responses to heat and a wide variety of other stressors. Increased levels of HSF1 facilitate survival in response to stress by coordinating a variety of fundamental cellular processes.^23^ Biochemical and genetic studies have clearly demonstrated critical roles for HSF1 in resistance to stress-induced inflammation and programmed cell death.^24^ Activation of HSF1 inhibits IL-1β-mediated inflammation, whereas the disruption of HSF1 augments proinflammatory TNF-α and IL-6 production in response to endotoxin stress.^25^ However, it is unknown how JAG1-mediated myeloid Notch signaling may interact with HSF1 in IR-stressed livers. We found that Notch1^FL/FL^ mice treated with JAG1 showed increased NICD and HSF1 levels, whereas Notch1^M-KO^ mice showed inhibited HSF1 expression, suggesting that JAG1-mediated Notch1 signaling mediates HSF1 activation. These data were consistent with a previous report that showed that Notch1 binds to the promoters of HSF1 to regulate the heat shock response pathway.^26^ Moreover, further evidence revealed that the adoptive transfer of BMMs pretreated with lentivirus expressing HSF1 into Notch1^M-KO^ mice ameliorated IR-induced hepatocellular damage. Thus, HSF1 is crucial for Notch1 signaling-mediated immune regulation in response to IR stress.

It is interesting to note that HSF1 induction inhibited ROS production, as evidenced by the reduced expression of 4-HNE, a key oxidative stress marker, in ischemic livers. Indeed, HNE activates stress response pathways, including antioxidant, endoplasmic reticulum stress, DNA damage, and heat shock response pathways.^27^ Notably, JAG1-mediated Notch1 signaling or HSF1 induction activated Snail, a critical gene for the control of cell movement and survival,^5^ whereas CRISPR/Cas9-mediated HSF1 knockout inhibited Snail while increasing NLRP3 and inflammatory cytokine expression. Moreover, although immunofluorescence staining revealed that JAG1 treatment increased Snail expression in macrophages, the disruption of HSF1 reduced Snail expression even during JAG1 treatment. These results suggest that HSF1 is essential for the activation of Snail, which may play an important regulatory role in Notch1 signaling-mediated immune regulation during liver IRI.

It is not known which other mechanisms may confer Snail with the ability to selectively affect NLRP3 activation during Notch1 signaling-mediated immune regulation. We found that the disruption of Snail with siRNA treatment aggravated IR-induced hepatocellular damage by increasing the expression of TXNIP, a critical signaling molecule involved in mediating NLRP3 activation.^28^ Indeed, TXNIP is an endogenous inhibitor and regulator of TRX. Enhanced TXNIP expression inhibits TRX activity and thus can modulate the cellular redox state and increase oxidative stress.^29^ Under the influence of ROS, TXNIP is translocated from the nucleus and binds to TRX1 in the cytosol, leading to increased ROS generation.^30^ Increased amounts of ROS can oxidize TRX1. The oxidized form of TRX1 is not able to bind to TXNIP, which is separated from TRX1. Free TXNIP physically interacts with the LRR domain of NLRP3 and activates NLRP3.^28^ Consistent with these results, TRX1 expression was markedly reduced in Snail siRNA-treated Notch1^FL/FL^ livers but not in NS siRNA-treated control livers after IR. Further evidence was produced by an in vitro study, which showed that the induction of Snail significantly increased TRX1 production but reduced TXNIP and ROS production, whereas the disruption of Snail increased ROS production and enhanced TXNIP/NLRP3 activation, resulting in increased caspase-1 activity and IL-1β release in LPS-stimulated macrophages. Thus, Snail regulates NLRP3 function through inhibiting ROS production and regulating the TXNIP/TRX1 complex in response to IR stress. Our findings demonstrate the unexpected role of myeloid Notch1 signaling-induced Snail activation in negatively modulating the NLRP3-mediated innate immune response during IR-triggered liver inflammation.

One striking finding was that myeloid Notch1-induced Snail activation could be involved in the modulation of apoptotic pathways during liver IRI. Indeed, both programmed cell death (apoptosis) and necrosis appear to be ongoing processes during liver IRI. Previous studies showed that TXNIP directly binds to TRX to inhibit its expression and activity.^31^ TRX has been identified as a binding partner of ASK1, a key regulator of oxidative stress-induced apoptosis in many types of cells through the activation of the JNK and p38 MAPK pathways.^32^ Under oxidative stress conditions, ROS induce the oxidation of TRX, leading to the dissociation of ASK1 from TRX and the activation of ASK1.^33^ In addition, TXNIP also binds and inhibits TRX, thus increasing the availability of activated ASK1.^34^ Thus, ASK1 could be critical to the Notch1/Snail-mediated regulation of the apoptotic process. As a result of the latter observation, we examined apoptotic liver cell death by TUNEL staining in vivo. JAG1 treatment of Notch1^FL/FL^ livers activated NICD and Snail, which was accompanied by a reduced number of apoptotic TUNEL^+^ cells after IR. However, disruption of Notch1 inhibited Snail activation, leading to markedly increased IR-induced cell apoptosis in Notch1^M-KO^ livers even during JAG1 treatment. Notably, enhanced ASK1 activation was found in Notch1^M-KO^ but not in Notch1^FL/FL^ livers. Moreover, using an in vitro macrophage/hepatocyte coculture system, we observed that the disruption of Snail in macrophages activated ASK1 in hepatocytes after coculture. Further evidence was produced by the immunofluorescence staining of hepatocytes after coculture with macrophages, which showed that the deletion of Snail in macrophages significantly increased the number of apoptotic TUNEL^+^ hepatocytes during H_2_O_2_-induced oxidative stress. Consistent with the in vivo study, JAG1 treatment increased antiapoptotic Bcl-2/Bcl-xL expression, whereas Snail deficiency in macrophages enhanced caspase-3 activation. Our findings demonstrate that Snail is a key regulator of ASK1-mediated hepatocyte apoptosis/necrosis in IR-stressed livers.

Another important implication of our results is that myeloid Notch1-induced Snail activation could also influence HMGB1 release during liver IRI. Indeed, HMGB1 can be released from macrophages and hepatocytes in response to oxidative stress. As an early mediator of inflammation, HMGB1 provides danger signals that activate immune cells and promote inflammatory responses and tissue damage during liver IRI. Inhibition of HMGB1 activity significantly diminished IR-induced hepatocellular damage.^35^ Although reduced serum levels of HMGB1 were observed in JAG1-treated Notch1^FL/FL^ mice, the disruption of myeloid Notch1 signaling augmented HMGB1 release in Notch1^M-KO^ mice. Because Snail is required for cell survival under oxidative stress conditions,^8^ it also appears to be involved in the modulation of HMGB1-mediated inflammation in liver IRI. Consistent with our in vitro data demonstrating the ability of Snail to inhibit HMGB1 induction in the coculture system, the disruption of Snail in macrophages increased HMGB1 release and hepatocyte death in H_2_O_2_-stressed hepatocytes. This result suggests that Snail may be crucial for the modulation of HMGB1-mediated inflammation and the reduction of hepatocellular damage in IR-triggered sterile liver inflammation. Taken together, our findings reveal the essential role of myeloid Notch-induced Snail activation in the regulation of apoptotic/necrotic pathways during liver IRI.

Figure 9 depicts the putative molecular mechanisms by which JAG1-mediated myeloid Notch1 signaling may regulate NLRP3 inflammasome-driven inflammatory responses in liver IRI. IR stress activates Notch1 signaling and induces ROS production in ischemic livers. Upon ligand binding, Notch1 is cleaved by γ-secretase, releasing the intracellular domain (NICD), which translocates into the nucleus and forms a complex with the CSL DNA-binding protein to activate HSF1; HSF1, in turn, promotes Snail activation and regulates the TRX1/TXNIP and TRX1/ASK1 complexes, leading to reduced NLRP3 inflammasome activation and ROS-induced hepatocellular apoptosis/necrosis during IR-triggered liver inflammation.

In conclusion, we demonstrated that JAG1-mediated myeloid Notch1 signaling promotes HSF1 and Snail activation, which in turn inhibit TXNIP/NLRP3 functioning and the apoptotic/necrotic pathway in IR-stressed livers. By identifying the molecular pathways by which JAG1-mediated myeloid Notch1/HSF1/Snail signaling regulates NLRP3-mediated innate immunity, our novel findings provide a rationale for the refinement of therapeutic approaches to ameliorate sterile inflammatory liver injury.

## Materials and methods

### Animals

Floxed Notch1 (Notch1^FL/FL^) mice (The Jackson Laboratory, Bar Harbor, ME) and mice expressing the Cre recombinase under the control of the lysozyme 2 (Lyz2) promoter (LysM-Cre; The Jackson Laboratory) were used to generate myeloid-specific Notch1 knockout (Notch1^M-KO^) mice, as described.^22^ Mouse genotyping was performed using a standard protocol with primers described in the JAX Genotyping protocols database. Animals at 6–8 weeks of age were used in all experiments. This study was performed in strict accordance with the recommendations in the Guide for the Care and Use of Laboratory Animals published by the National Institutes of Health. The study protocols were approved by the Institutional Animal Care and Use Committee of The University of California, Los Angeles and Shanghai Jiaotong University in China.

### Mouse liver IRI model

We used an established mouse model that utilized warm hepatic ischemia followed by reperfusion.^2^ Mice were injected with heparin (100 U/kg), and an atraumatic clip was used to interrupt the arterial/portal venous blood supply to the cephalad liver lobes. After 90 min of ischemia, the clip was removed, and mice were sacrificed after 6 h of reperfusion. Some animals were injected via the tail vein with bone marrow-derived macrophages (BMMs, 5 × 10^6^ cells in PBS/mouse) transfected with lentivirus-expressing HSF1 (Lv-HSF1) 24 h prior to ischemia, and some animals were injected i.v. with Snail siRNA or nonspecific (control) siRNA (2 mg/kg) (Santa Cruz Biotechnology, CA) mixed with mannose-conjugated polymers (Polyplus transfection™, Illkirch, France) at a ratio defined according to the manufacturer’s instructions 4 h prior to ischemia, as described.^2^ Some animals were injected i.v. with recombinant JAG1 (0.5 mg/kg, R&D Systems) or PBS 1 h prior to ischemia.

### Hepatocellular function assay

The levels of serum alanine aminotransferase (sALT), an indicator of hepatocellular injury, were measured by IDEXX Laboratories (Westbrook, ME).

### Histology, immunohistochemistry, and immunofluorescence staining

Liver sections (5 μm) were stained with hematoxylin and eosin (H&E). The severity of IRI was graded using Suzuki’s criteria^36^ on a scale from 0 to 4. Liver macrophages were detected using rat anti-mouse CD11b Ab (BD Biosciences) as the primary antibody and Alexa Fluor 594 anti-rat IgG as the secondary antibody for immunofluorescence staining. DAPI was used for nuclear counterstaining. Liver neutrophils were detected using primary rat anti-mouse Ly6G Ab (BD Biosciences). Biotinylated goat anti-rat IgG (Vector, Burlingame, CA) was used as a secondary antibody for immunohistochemistry staining. Snail in macrophages was detected using rabbit anti-mouse Snail Ab (Cell Signaling Technology, MA) as a primary antibody and Alexa Fluor-conjugated AffiniPure donkey anti-rabbit IgG Ab (Jackson Immunoresearch, PA) as a secondary antibody, and macrophages were incubated with immunoperoxidase (ABC Kit, Vector) according to the manufacturer’s instructions. The positive cells were counted blindly at 10 HPF/section (200×).

### Myeloperoxidase activity assay

The presence of myeloperoxidase (MPO) was used as an index of hepatic neutrophil accumulation.^2^ The change in absorbance was measured spectrophotometrically at 655 nm. One unit of MPO activity was defined as the quantity of enzyme that degraded 1 μmol peroxide/min at 25 °C per gram of tissue.

### TUNEL assay

The Klenow-FragEL DNA Fragmentation Detection Kit (EMD Chemicals, Gibbstown, NJ) was used to detect DNA fragmentation characteristic of oncosis/ apoptosis/necrosis in formalin-fixed paraffin-embedded liver sections.^22^ The apoptosis of primary hepatocytes was measured by using a Cell Meter TUNEL Apoptosis Assay Kit (AAT Bioquest, Sunnyvale, CA). Briefly, treated hepatocytes were fixed in 4% paraformaldehyde for 30 min. After three washes with TBST, the hepatocytes were incubated with Tunnelyte^TM^ Green for 60 min at 37 °C. Additional Hoechst staining was conducted for nucleus identification. The TUNEL-positive cells were visually identified by fluorescence microscopy by using an FITC filter. The results were scored semiquantitatively by averaging the number of apoptotic cells/microscopic field at ×200 magnification. Ten fields were evaluated per sample.

### Quantitative RT-PCR analysis

Quantitative real-time PCR was performed as previously described.^2^ Total RNA was purified from liver tissue or cell cultures using the RNeasy Mini Kit (Qiagen, Chatsworth, CA) according to the manufacturer’s instructions. Reverse transcription to generate cDNA was performed by using the SuperScript III First Strand Synthesis System (Invitrogen). Quantitative real-time PCR was performed using the DNA Engine with Chromo 4 Detector (MJ Research, Waltham, MA). To a final reaction volume of 25 μl, the following were added: 1× SuperMix (Platinum SYBR Green qPCR Kit; Invitrogen, San Diego, CA), cDNA and 10 μM of each primer. The amplification conditions were as follows: 50 °C (2 min) and 95 °C (5 min) followed by 40 cycles of 95 °C (15 s) and 60 °C (30 s). The primer sequences used for the amplification of TNF-α, IL-1β, MCP-1, CXCL-1, CXCL-2, CXCL-10, and HPRT are shown in Supplementary Table 1. Target gene expression was calculated according to the ratio of target gene expression to that of the housekeeping gene HPRT.

### Western blot analysis

Protein was extracted from liver tissue or cell cultures as described.^2^ Protein was extracted from liver tissue or cell cultures with ice-cold protein lysis buffer (50 mM Tris, 150 mM NaCl, 0.1% sodium dodecyl sulfate, 1% sodium deoxycholate, and 1% Triton-100). Proteins (30 µg/sample) in SDS-loading buffer (50 mM Tris, pH 7.6, 10% glycerol, and 1% SDS) were subjected to 4–20% SDS-polyacrylamide gel electrophoresis (PAGE) and transferred to a nitrocellulose membrane (Bio-Rad, Hercules, CA). The membrane was blocked with 5% dry milk and 0.1% Tween 20 (USB, Cleveland, OH). Monoclonal rabbit anti-mouse NICD, HSF1, Snail, TRX1, TXNIP, NLRP3, ASC, cleaved caspase-1, p-ASK1, ASK1, Bcl-2, Bcl-xL, cleaved caspase-3, and β-actin Abs (Cell Signaling Technology, MA) were used. The membranes were incubated with the Abs and then developed according to the Pierce SuperSignal West Pico Chemiluminescent Substrate protocol (Pierce Biotechnology, Rockford, IL). The relative quantities of protein were determined using a densitometer (Kodak Digital Science 1D Analysis Software, Rochester, NY) and expressed in absorbance units (AU) by comparison with β-actin expression.

### Lentiviral vector construction

The HSF1 lentiviral vector (p-Lv-HSF1, Applied Biological Materials Inc., Canada), which expresses HSF1 and contains a CMV promoter and a kanamycin gene, was packaged with psPAX2 and pVSVG (Addgene). The 293T cell line was cultured in Dulbecco’s modified Eagle medium (DMEM) supplemented with 10% fetal bovine serum (FBS). Cells were seeded in six-well plates and transfected when they reached 60–70% confluence. Cells were cotransfected with p-Lv-HSF1, psPAX2 and pVSVG using Lipofectamine 3000 reagent (Invitrogen) to package the lentiviruses according to the manufacturer’s instructions. The following amounts of plasmid DNA were used per well: 1 μg of p-Lv-HSF1 (or control vector), 0.625 μg of psPAX2, and 0.375 μg of pCMV-VSVG. Forty-eight hours after transfection, the viral vector-containing supernatant was collected and filtered through a 0.45 μm filter. The HSF1 lentivirus (Lv-HSF1) was either used immediately or snap-frozen at −80 °C for later use. The GFP lentivirus (Lv-GFP, Applied Biological Materials Inc.) was used as a control.

### Primary hepatocyte/BMM isolation and in vitro transfection

Murine primary hepatocytes and bone-derived macrophages (BMMs) were isolated from Notch1^FL/FL^ and Notch1^M-KO^ mice as described.^2^ In brief, livers were perfused in situ with warmed (37˚C) HBSS solution followed by collagenase buffer (collagenase type IV, Sigma, St Louis, MO). The perfused livers were dissected and strained through 70-μm nylon mesh cell strainers (BD Biosciences). The nonparenchymal cells (NPCs) were separated from the hepatocytes by centrifugation three times at 50 × g for 2 min. The NPCs were suspended in HBSS and layered onto a 50%/25% two-step Percoll gradient (Sigma) in a 50-ml conical centrifuge tube and centrifuged at 1800 × *g* at 4 °C for 15 min. Bone marrow cells were removed from the femurs and tibias of the Notch1^FL/FL^ and Notch1^M-KO^ mice and cultured in DMEM supplemented with 10% FBS and 15% L929-conditioned medium. BMMs (1 × 10^6^/well) were cultured for 7 days and then transfected with CRISPR/Cas9-mediated HSF1 KO (p-CRISPR-HSF1 KO), CRISPR/Cas9-mediated Snail KO (p-CRISPR-Snail KO), CRISPR/Cas9-mediated Snail activation (p-CRISPR-Snail) or control vector. After 48 h, the cells were treated with 2 µg/ml JAG1 supplemented with 100 ng/ml LPS for an additional 6 h.

### Coculture of macrophages and primary hepatocytes

Primary hepatocytes were cultured in six-well plates at a concentration of 4 × 10^5^ cells per well. After 24 h, 0.4 μm-pore size Transwell inserts (Corning) containing 1 × 10^6^ BMMs were placed into the six-well plates with the hepatocytes that were initially seeded. The cocultures were incubated for 12 h with or without the addition of H_2_O_2_ (200 µM) to the lower chamber.

### ELISA assay

Murine serum and cell culture supernatants were harvested for cytokine analysis. ELISA kits were used to measure the TNF-α, IL-1β, and HMGB1 levels (eBiosciences).

### LDH activity assay

BMMs (1 × 10^6^) were cultured with primary hepatocytes (4 × 10^5^/well) for 12 h with or without the addition of H_2_O_2_ (200 µM) to the lower chamber. The activity of lactate dehydrogenase (LDH) in the cell culture medium from the lower chamber was measured with a commercial LDH activity assay kit (Stanbio Laboratory, Boerne, TX) according to the manufacturer’s instructions.

### Reactive oxygen species assay

ROS production in BMMs was measured using 5- and 6-carboxy-2′,7′-difluorodihydrofluorescein diacetate (Carboxy-H2DFFDA, Life Technologies) as described.^2^ In brief, BMMs from Notch1^FL/FL^ and Notch1^M-KO^ mice were transfected with CRISPR/Cas9-Snail KO, CRISPR/Cas9-Snail activation or control vector and then cultured on collagen-coated coverslips after LPS stimulation. After washing with PBS, the cells were incubated with 10 μM of Carboxy-H2DFFDA. Carboxy-H2DFFDA was converted to a fluorescent green form when it was hydrolyzed by intracellular esterases and oxidized in cells. The cells were then fixed with 2% paraformaldehyde and stained with Hoechst dye. The ROS produced by the BMMs was analyzed and quantified by fluorescence microscopy. Positive green fluorescent-labeled cells were counted blindly at 10 HPF/section (200×).

### Caspase-1 enzymatic activity assay

Caspase-1 enzymatic activity was determined by a colorimetric assay kit (R&D System), as described.^37^ Briefly, after coculture with MSCs, 50 µl of cell lysate from BMMs was added to 50 µl of caspase-1 reaction buffer in a 96-well flat bottom microplate. Each sample was then added to 200 mM caspase-1 substrate and WEHD-pNA, followed by 2 h of incubation at 37 °C. The enzymatic activity of caspase-1 was measured with an ELISA reader at a wavelength of 405 nm.

### Statistical analysis

Data are expressed as the mean ± SD and were analyzed by the permutation *t* test and Pearson correlation. Two-sided *p* values less than 0.05 were considered statistically significant. Multiple group comparisons were made using one-way ANOVA followed by Bonferroni’s post hoc test. When the groups showed unequal variances, we applied Welch’s ANOVA to generate the multiple group comparisons. All analyses were performed by using SAS/STAT software, version 9.4.

## Supplementary information

Supplementary Table 1

## References

[1] Lentsch AB, Kato A, Yoshidome H, McMasters KM, Edwards MJ (2000). Inflammatory mechanisms and therapeutic strategies for warm hepatic ischemia/reperfusion injury. Hepatology.

[2] Yue S (2016). The myeloid heat shock transcription factor 1/beta-catenin axis regulates NLR family, pyrin domain-containing 3 inflammasome activation in mouse liver ischemia/reperfusion injury. Hepatology.

[3] Nieto MA (2002). The snail superfamily of zinc-finger transcription factors. Nat. Rev. Mol. Cell Biol..

[4] Carver EA, Jiang R, Lan Y, Oram KF, Gridley T (2001). The mouse snail gene encodes a key regulator of the epithelial−mesenchymal transition. Mol. Cell Biol..

[5] Barrallo-Gimeno A, Nieto MA (2005). The Snail genes as inducers of cell movement and survival: implications in development and cancer. Development.

[6] Kudo-Saito C, Shirako H, Takeuchi T, Kawakami Y (2009). Cancer metastasis is accelerated through immunosuppression during Snail-induced EMT of cancer cells. Cancer Cell.

[7] Hotz B, Visekruna A, Buhr HJ, Hotz HG (2010). Beyond epithelial to mesenchymal transition: a novel role for the transcription factor Snail in inflammation and wound healing. J. Gastrointest. Surg..

[8] Kim NH (2017). Snail reprograms glucose metabolism by repressing phosphofructokinase PFKP allowing cancer cell survival under metabolic stress. Nat. Commun..

[9] Mumm JS, Kopan R (2000). Notch signaling: from the outside in. Dev. Biol..

[10] Kopan R, Ilagan MX (2009). The canonical Notch signaling pathway: unfolding the activation mechanism. Cell.

[11] Shimizu K, Chiba S, Saito T, Kumano K, Hirai H (2000). Physical interaction of Delta1, Jagged1, and Jagged2 with Notch1 and Notch3 receptors. Biochem. Biophys. Res. Commun..

[12] Manderfield LJ (2012). Notch activation of Jagged1 contributes to the assembly of the arterial wall. Circulation.

[13] Kohler C (2004). Expression of Notch-1 and its ligand Jagged-1 in rat liver during liver regeneration. Hepatology.

[14] Radtke F, MacDonald HR, Tacchini-Cottier F (2013). Regulation of innate and adaptive immunity by Notch. Nat. Rev. Immunol..

[15] Yu HC (2011). Canonical notch pathway protects hepatocytes from ischemia/reperfusion injury in mice by repressing reactive oxygen species production through JAK2/STAT3 signaling. Hepatology.

[16] Liou GY, Storz P (2015). Detecting reactive oxygen species by immunohistochemistry. Methods Mol. Biol..

[17] Palaga T (2008). Notch signaling is activated by TLR stimulation and regulates macrophage functions. Eur. J. Immunol..

[18] Ostroukhova M (2006). Treg-mediated immunosuppression involves activation of the Notch-HES1 axis by membrane-bound TGF-beta. J. Clin. Invest..

[19] Zhang Q (2012). Notch signal suppresses Toll-like receptor-triggered inflammatory responses in macrophages by inhibiting extracellular signal-regulated kinase 1/2-mediated nuclear factor kappaB activation. J. Biol. Chem..

[20] Hu X (2008). Integrated regulation of Toll-like receptor responses by Notch and interferon-gamma pathways. Immunity.

[21] Shang Y (2016). The transcriptional repressor Hes1 attenuates inflammation by regulating transcription elongation. Nat. Immunol..

[22] Lu L (2018). Myeloid Notch1 deficiency activates the RhoA/ROCK pathway and aggravates hepatocellular damage in mouse ischemic livers. Hepatology.

[23] Dai C, Whitesell L, Rogers AB, Lindquist S (2007). Heat shock factor 1 is a powerful multifaceted modifier of carcinogenesis. Cell.

[24] Tanaka K (2007). Genetic evidence for a protective role for heat shock factor 1 and heat shock protein 70 against colitis. J. Biol. Chem..

[25] Xie Y, Chen C, Stevenson MA, Auron PE, Calderwood SK (2002). Heat shock factor 1 represses transcription of the IL-1beta gene through physical interaction with the nuclear factor of interleukin 6. J. Biol. Chem..

[26] Kourtis N (2018). Oncogenic hijacking of the stress response machinery in T cell acute lymphoblastic leukemia. Nat. Med..

[27] West JD, Marnett LJ (2005). Alterations in gene expression induced by the lipid peroxidation product, 4-hydroxy-2-nonenal. Chem. Res. Toxicol..

[28] Zhou R, Tardivel A, Thorens B, Choi I, Tschopp J (2010). Thioredoxin-interacting protein links oxidative stress to inflammasome activation. Nat. Immunol..

[29] Spindel ON, World C, Berk BC (2012). Thioredoxin interacting protein: redox dependent and independent regulatory mechanisms. Antioxid. Redox Signal..

[30] Harijith A, Ebenezer DL, Natarajan V (2014). Reactive oxygen species at the crossroads of inflammasome and inflammation. Front. Physiol..

[31] Nishiyama A (1999). Identification of thioredoxin-binding protein-2/vitamin D(3) up-regulated protein 1 as a negative regulator of thioredoxin function and expression. J. Biol. Chem..

[32] Tobiume K (2001). ASK1 is required for sustained activations of JNK/p38 MAP kinases and apoptosis. EMBO Rep..

[33] Saitoh M (1998). Mammalian thioredoxin is a direct inhibitor of apoptosis signal-regulating kinase (ASK) 1. EMBO J..

[34] Zhou J, Yu Q, Chng WJ (2011). TXNIP (VDUP-1, TBP-2): a major redox regulator commonly suppressed in cancer by epigenetic mechanisms. Int. J. Biochem. Cell Biol..

[35] Tsung A (2005). The nuclear factor HMGB1 mediates hepatic injury after murine liver ischemia-reperfusion. J. Exp. Med..

[36] Suzuki S, Toledo-Pereyra LH, Rodriguez FJ, Cejalvo D (1993). Neutrophil infiltration as an important factor in liver ischemia and reperfusion injury. Modulating effects of FK506 and cyclosporine. Transplantation.

[37] Kamo N (2013). ASC/caspase-1/IL-1beta signaling triggers inflammatory responses by promoting HMGB1 induction in liver ischemia/reperfusion injury. Hepatology.

