# Supplementary material for: Jagged1-mediated myeloid Notch1 signaling activates HSF1/Snail and controls NLRP3 inflammasome activation in liver inflammatory injury
Source: Cell Mol Immunol. 2019 Oct 31;17(12):1245–56. doi: 10.1038/s41423-019-0318-x (PMC7784844; doi:10.1038/s41423-019-0318-x)
Supplement: Supplementary file 1 — Supplementary Table 1 [file 41423_2019_318_MOESM1_ESM.docx]

Supplementary Table 1

| Target genes | Forward primers | Reverse primers |
| --- | --- | --- |
| HPRT | 5’-TCAACGGGGGACATAAAAGT-3’ | 5’-TGCATTGTTTTACCAGTGTCAA-3’ |
| TNF-α | 5’-GCCTCTTCTCATTCCTGCTTGT-3’ | 5’-GATGATCTGAGTGTGAGGGTCTG-3’ |
| IL-1β | 5’-TGTAATGAAAGACGGCACACC-3’ | 5’-TCTTCTTTGGGTATTGCTTGG-3’ |
| MCP-1 | 5'-GAAGGAATGGGTCCAGACAT-3' | 5'-ACGGGTCAACTTCACATTCA-3' |
| CXCL-2 | 5'-ACTTCAAGAACATCCAGAG-3' | 5'-CTTTCCAGGTCAGTTAGC-3' |
| CXCL-10 | 5’-GCTGCCGTCATTTTCTGC-3’ | 5’-TCTCACTGGCCCGTCATC-3’ |
| CXCL-1 | 5’-TGGCTGGGATTCACCTCAAGAACA-3’ | 5’-TTTCTGAACCAAGGGAGCTTCAGG-3’ |
